# Occurrence of Treehoppers (Hemiptera: Membracidae: Smiliinae) on Oaks in Delaware Water Gap National Recreation Area, 2004–2006

**DOI:** 10.1673/031.008.5901

**Published:** 2008-10-16

**Authors:** Matthew S. Wallace

**Affiliations:** Department of Biological Sciences, East Stroudsburg University, 200 Prospect Street, East Stroudsburg, PA 18301-2999

**Keywords:** Homoptera, Auchenorrhyncha, sampling, sticky cards, host plants

## Abstract

A total of 870 treehoppers and 24 species from the tribe Smiliini (Hemiptera: Membracidae: Smiliinae) were collected from various oaks in the Delaware Water Gap National Recreation Area in 2006 using yellow sticky cards. Combining all years and collecting methods, 27 species were found in the park. A majority of the specimens collected in 2006 were males of *Cyrtolobus vau* and *Ophiderma pubescens*, as in previous years. Most of the treehoppers were caught in mid to late June, comparable to 2004 and 2005. It appears that many species are segregated either temporally or by oak group; some treehopper species show preference for either the red or white oak group rather than for one species of oak. Color photographs for 27 treehopper species (many including both sexes) are included.

## Introduction

Treehoppers, or membracids, feed on phloem of many plant species. Oaks (Fagales: Fagaceae: *Quercus*) are the most prominent hosts of treehoppers in the eastern United States. These insects are characterized by an enlarged pronotum that can resemble various plant structures such as buds, leaves, and thorns. Although treehoppers are not considered serious agricultural or forest pests, some mechanically injure plant stems during oviposition (Wallace and Deitz 2007). Recent research on treehoppers includes works on their systematics (Wallace and Deitz 2004; Cryan et al. 2004; Dietrich 2005); ecology and behavior including parental care, ant-mutualism, and acoustic communication (Wood 1993; Cocroft et al. 2006; Morales and Beal 2006); and biodiversity, life history, and interactions with plants (Wood 1993; Wallace and Troyano 2006). Several workers (Mason and Loye 1981; Johnson and Freytag 1997; Dietrich et al. 1999; Wallace et al. 2003; Wallace and Troyano 2006; and Bartlett et al. 2008) have examined treehopper species diversity and their host associations in different areas of the eastern U.S. Funkhouser's (1917) “Biology of the Membracidae of the Cayuga Lake Basin” remains the largest and most important work on Nearctic treehopper biology. Membracid ecology in Pennsylvania has been investigated by Moul (1943), Frost (1955, 1957), and Wallace and Troyano (2006). Approximately 97 membracid species have been documented from Pennsylvania.

Recent studies highlight the need for conservation of treehoppers and their kin. Despite their potential as pests, treehoppers are likely beneficial insects in certain situations. Styrsky and Eubanks (2007) suggested that some plants may indirectly benefit from the mutualistic association between treehoppers and their ant attendees. Predaceous ants may discourage outbreaks of pest herbivores on plants that ants inhabit. Furthermore, treehoppers are common herbivores in many ecosystems and are prey items for vertebrate and invertebrate predators. Treehopper populations are now threatened by many factors. The health of eastern forests of the United States continues to be endangered by exotic organisms and abiotic factors such as increasing levels of air pollution. The spread of urban growth and the destruction of forested areas also jeopardize eastern deciduous forests. Oaks, and therefore treehoppers and other oak herbivores, are threatened by diseases such as sudden oak death (O'Brien et al. 2002) and bacterial leaf scorch (Lashomb et al. 2002). Indeed, treehoppers have been implicated as potential vectors of bacterial leaf scorch (Lashomb et al. 2002).

A major roadblock to insect conservation worldwide is the lack of knowledge of insect life histories and the paucity of published species-lists in threatened ecosystems. Despite the recent work on treehopper biology, there is still much to learn about treehopper diversity and their host specificity with respect to oaks in most ecosystems of the eastern United States. The aforementioned recent findings call attention to the need for a better understanding of treehopper biological diversity and host associations.

Wallace and Troyano (2006) reported treehopper diversity and seasonal abundance from the Pennsylvania side of Delaware Water Gap National Recreation Area (DWGNRA) in 2004 and 2005. DWGNRA is a popular national tourist site located along the upper Delaware River in eastern Pennsylvania and western New Jersey. The final year of the project (2006) yielded an unexpectedly large number of treehopper specimens and taxa. Indeed, more than three times as many specimens were caught in 2006 than in 2004 and 2005 combined. The results from 2006 as well as summary information on treehopper host associations and seasonal abundance from all 3 years are presented here. Most of the eastern North American membracids are in the subfamily Smiliinae. This research focuses on the diversity of the tribe Smiliini in DWGNRA of eastern Pennsylvania. The Smiliini (Hemiptera: Membracidae: Smiliinae) include the following Nearctic, primarily oak-feeding genera: *Archasia, Atymna, Cyrtolobus, Glossonotus, Heliria, Helonica, Ophiderma, Palonica, Smilia, Telamona*, and *Xantholobus*.

The present research provides basic biological data on treehoppers including the species present, their host associations, and abundance that will serve as a benchmark for biodiversity in DWGNRA and eastern Pennsylvania. Because treehoppers are so closely allied with the dominant plant members of the eastern forest, they, along with their hosts, may serve as important biological indicators of ecosystem health.

## Materials and Methods

Methods used in 2006 closely followed those used in 2004 and 2005 (Wallace and Troyano 2006). Sites were chosen based on 3 years of cumulative scouting for the best treehopper collecting areas. Treehoppers are usually abundant on trees at the forest's edge or in disturbed areas, with limb tips exposed to the sun. An attempt was made to select trees that were isolated from other oak species to help assure that captured treehoppers came from the chosen tree. Only branches reachable by hand were sampled for treehoppers.

**Figure 1.**
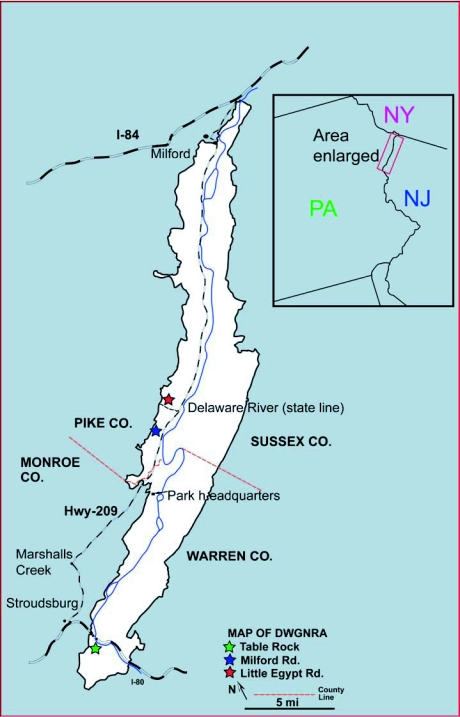
Map of study trees in Delaware Water Gap National Recreation Area, 2006.

Thirty oak trees were selected for sampling from three sites on the Pennsylvania side of the Park in early 2006 (Figure 1). Oak species included 3 from the “red oak” group: black (*Quercus velutina* Lam., 6 specimens), scarlet (*Q. coccinea* Muenchh., 3 specimens), and red (*Q. rubra* L., 7 specimens); and 2 from the “white oak” group: chestnut (*Q. prinus* L., 7 specimens) and white (*Q. alba* L., 7 specimens).

The coordinates and number of trees sampled for the 3 sites are as follows: Table Rock (9 trees), N40°58.49′ W75°08.50′; Milford Road (9 trees), N41°06.99′ W74°59.53′; and Little Egypt Road (12 trees), N41°08.11 W74°58.16′. Table Rock is approximately 32 km south of Milford Road and Little Egypt Road, which are only several km apart (Figure 1). Elevations for the sites are: Table Rock, 183–198 m; Milford Rd., 213 m; and Little Egypt Rd., 244 m. The Table Rock site lies along a ridge and consists of numerous exposed rock slopes dominated by small chestnut oak trees. Red oak was also sampled at this site. The Milford Road site is an abandoned field with black, scarlet, and white oak trees at the border of the field and the forest. Little Egypt Road is in the forest but was recently disturbed, leaving many open areas. This site was also sampled in both 2004 and 2005 (Wallace and Troyano 2006). Red, scarlet, and white oaks were used at this site.

To collect treehoppers, 3 × 5 inch yellow sticky card traps (Olson Products Sticky Strips, www.olsonproducts.com) were suspended from a lower canopy branch of each study tree using twist ties. Previous research has shown that yellow sticky card traps are effective in catching adult membracids (Mason and Loye 1981; Johnson and Freytag 1997; Wallace and Troyano 2006). Sampling was performed to coincide with eastern treehopper adult phenology based on existing host data and past studies (Wallace and Troyano 2006). In 2005, adult treehoppers first appeared on sticky cards in late May, approximately 5 weeks after oak bud break in late April. In 2006, trapping began in early May based on observed oak budbreak (approximately 17–21 April 2006 in DWGNRA). Sampling began in early May, before adult treehoppers were expected, in order to establish a baseline for phenology studies. Traps were collected and replaced with a fresh trap weekly (for most periods) starting 9 May and ending 8 August for a total of 12 collection periods. The last collection period (25 July-8 August) was the only two-week sample. Pinned specimens have been deposited at East Stroudsburg University, East Stroudsburg, PA. Several times during the 2006 season yellow sticky cards were found on the ground near the study tree or not found at all, but their loss was not considered to impact the results.

Summary data on seasonal abundance and host associations using yellow sticky cards were tabulated using data from 2004–2006 (Wallace and Troyano 2006). Adult treehoppers were also collected periodically during the 3-year period in the park by sweeping oaks and by removing individuals by hand, and their host associations are included in the summary table. Since the goal of this study was to trap adults and not nymphs, oak species from which adult treehoppers were collected are here termed host associations, and not host plants.

Treehoppers were identified by matching captured specimens to authoritatively identified museum specimens and by comparing captured specimens to specimen drawings in Woodruff (1924) and Kopp and Yonke (1973, 1974).

## Results

A total of 870 treehopper specimens in 24 species from the tribe Smiliini known to associate with oak were captured using yellow sticky cards in DWGNRA in 2006 (Table 1). This includes 1 unidentified specimen and 17 specimens with dubious identifications, most involving uncertain identifications of *Cyrtolobus discoidalis* and *Xantholobus intermedius.* The males of these two species are very difficult to distinguish from each other, and none of the specimens collected match precisely published illustrations and descriptions (Woodruff 1924; Kopp and Yonke 1973, 1974).

A total of 24 species (excluding doubted identifications) in 8 genera were trapped (Table 1). The most numerous species collected (about 35% of the total specimens) was *Cyrtolobus vau* (n = 306). Other notable taxa were *Ophiderma pubescens* (about 23% of the total, n = 199), *O. definita* (n = 64), *Glossonotus univittatus* (n=58), and *Telamona decorata* (n=48). The most abundant genus in terms of specimens collected was *Cyrtolobus* (n = 360, about 41% of total). *Cyrtolobus* was also the richest in numbers of species collected, accounting for 9 of the 24 species that had been authoritatively identified.

**Figure 2.**
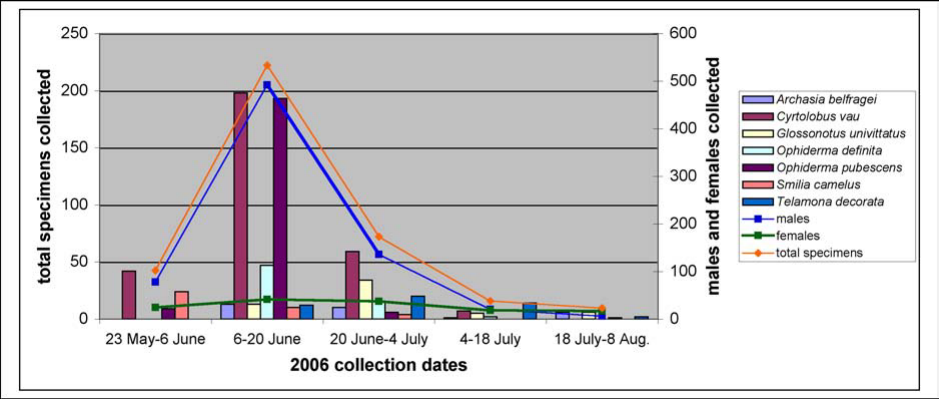
Seasonal abundance of all oak-feeding smiliine treehoppers collected, the 7 most abundant species, and male and female abundance in DWGNRA (2006). Although in most cases traps were collected and replaced weekly, for purposes of presentation, they were combined into two-week intervals here.

The total number of trapped adult treehoppers (sites combined) peaked during the week of 13–20 June (Figure 2). The first specimens were captured during the week of 23–30 May and specimen numbers steadily rose until the peak week. The seven most numerous species were trapped at various times during the season (Figure 2). *Smilia camelus* was most frequently collected in late May-early June. The highest numbers of *Archasia belfragei, Cyrtolobus vau, Ophiderma definita*, and *O. pubescens* were trapped in mid-June, while *Glossonotus univittatus* and *Telamona decorata* were most frequently present in late June-early July. The number of males collected in May and June of 2006 greatly outnumbered the number of females collected (Figure 2). However, in July and August more females were collected than males. A total of 733 males and 136 females were collected (1 unidentified specimen could not be sexed).

Collection dates for all species caught on sticky cards from 2004–2006 are shown in Table 2. Species collected most frequently from late May to early June (early season) included *Atymna querci, Cyrtolobus tuberosus, Ophiderma salamandra*, and *S. camelus*. Treehoppers most common during all 3 years in mid-June were *A. belfragei, C. dixianus, C. pallidifrontis, C. vau, Glossonotus acuminatus, O. definita, O. flavicephala, O. pubescens*, and *Telamona monticola*. During late-June to early July, the most frequent species collected were *C. fenestratus, C. fuscipennis, C. puritanus, G. univittatus*, and *T. decorata*. The only species to show discernible peaks from late July to August were *T. compacta* and *T. reclivata*. See Figures 3–44 for photographs of selected species.

During the 3-year collection period, treehoppers were collected from many oak species (Table 3). Most specimens were collected from chestnut oak (345), red oak (307), and white oak (276). The most species were collected from white (21), followed by red (17), and chestnut (17). All but one treehopper species, *C. dixianus*, was collected from multiple oak species during the 3-year period (when including only taxa with greater than 5 specimens collected). *C. dixianus* was only collected on chestnut oak. Several species were more commonly collected on trees in the “white oak group” (chestnut and white) compared to the “red oak group” (black, pin, red, scarlet, and scrub), and vice-versa (when including only taxa with greater than 5 specimens collected). Those most common on the white oak group were: *A. querci* (100% on white oak group), *C. pallidifrontis* (95%), *C. tuberosus* (89%), *C. vau* (90%), *T. decorata* (98%), *T. monticola* (89%), and *T. reclivata* (90%), while those more common on the red oak group were *C. puritanus* (78% on red oak group), *O. definita* (84%), *O. flavicephala* (78%), *O. pubescens* (94%), *O. salamandra* (100%), and *S. camelus* (76%). *G. univittatus* (60% on white oak group) showed no strong host association with either group. In the first two years of the study, some treehoppers were also captured at selected areas in the Park by hand and sweeping from oak trees. All species collected by this method were also collected in the sticky card study (Table 3).

**Table 1.**
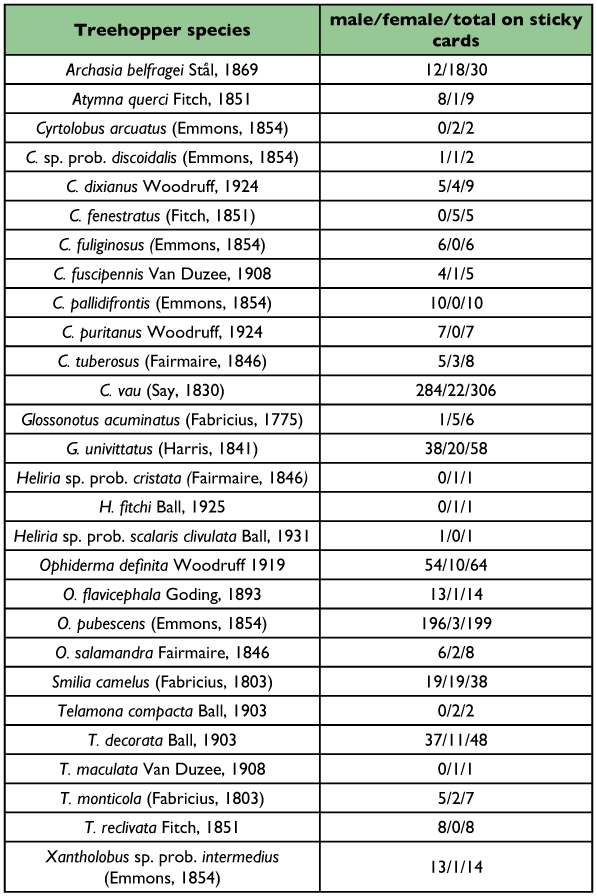
Identified oak-feeding smiliine treehoppers collected from DWGNRA (2006).

## Discussion

This was the final year of a 3-year project. Results of the first two years (2004–2005) were summarized by Wallace and Troyano (2006). More than three times as many specimens were caught in 2006 than in 2004 and 2005 combined despite sampling for 2 less weeks in 2006 than 2005. This disparity in specimen number is likely due to the sites chosen in 2007. All three sites had plentiful oaks with lower branches exposed to sunlight, a condition favorable for treehoppers. Furthermore, a larger number of trees were used at the Little Egypt Road site in 2006 (n = 12) compared to 2005 (n = 4). This area has always been productive throughout the study, likely due to the moderate disturbance of the site. Large numbers of specimens and species were also caught at Table Rock. This may be partially explained by the numerous exposed rock slopes at this site, which may absorb heat during the day and stay warm during the night, creating a warmer environment for the insects to live compared to the other sites that had no exposed rock faces. Other insects such as dipterans and hymenopterans are known to favor similar habitats, such as mountain summits (Chapman 1954).

**Table 2.**
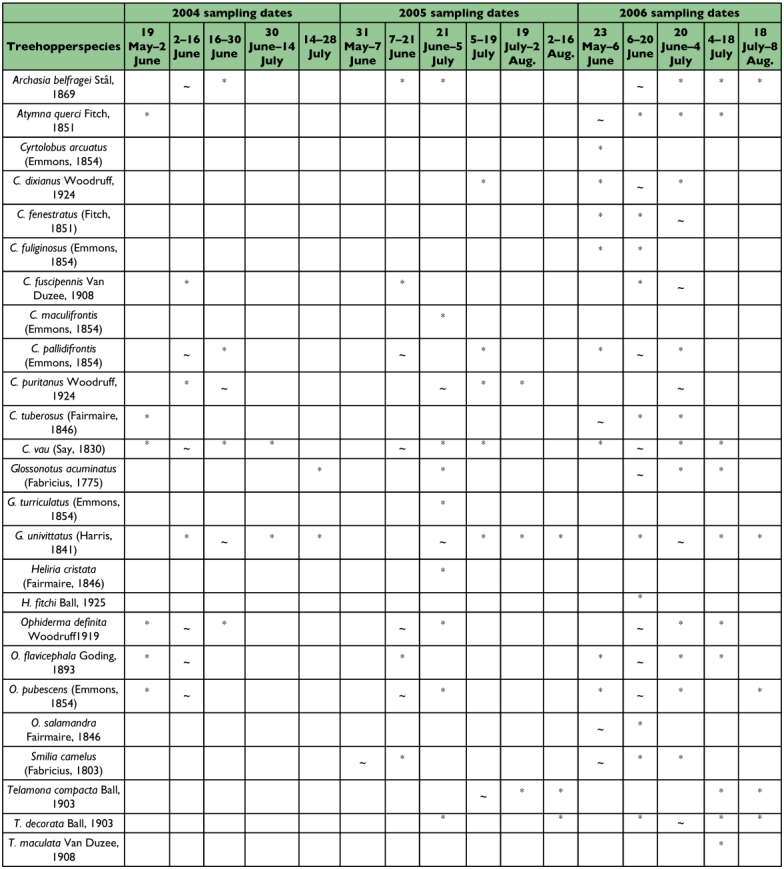
Collection dates for identifed treehoppers from DWGNRA (2004–2006) using yellow sticky cards. Some of these data were published by Wallace and Troyano (2006) and are included here for comparison.

**con't:**
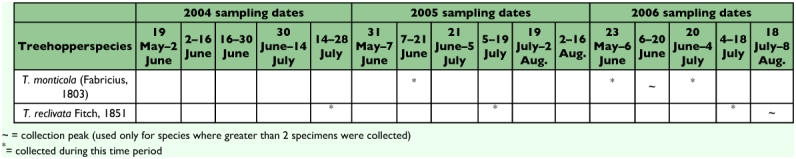

Combining all 3 years, a total of 27 treehopper species from the tribe Smiliini (excluding doubtful identifications) associated with oak were collected by sticky traps, sweeping, and hand-picking. The species of treehoppers collected in 2006 (Tables 1, 3) were similar to those caught in 2004 and 2005. Notable exceptions include *C. fenestratus, C. arcuatus, O. salamandra*, and *Telamona maculata*, which were not caught in the previous 2 years of the study. Nevertheless, *Cyrtolobus maculifrontis* and *Glossonotus turriculatus* were caught in 2004 and 2005 but not in 2006.

The four most commonly collected smiliine genera in 2004–2005 (Wallace and Troyano 2006)--*Cyrtolobus, Ophiderma, Glossonotus*, and *Telamona*--were also the most dominant in 2006 (Table 1, Figure 2). Similarly, the most commonly encountered genera in terms of species and specimens were *Cyrtolobus* and *Ophiderma* (Table 1, Figure 2). *C. vau* and *O. pubescens* were the most common species caught on sticky cards throughout the study, and it appears that these species may be the dominate treehoppers in oak forests in eastern Pennsylvania. Frost (1957) collected more *C. vau* than any other species at light traps in early July in Centre County, Pennsylvania. The biology and life history of *C. vau* and *O. pubescens* may merit further study if treehoppers are found to be vectors of oak pathogens.

**Figures 3–8.**
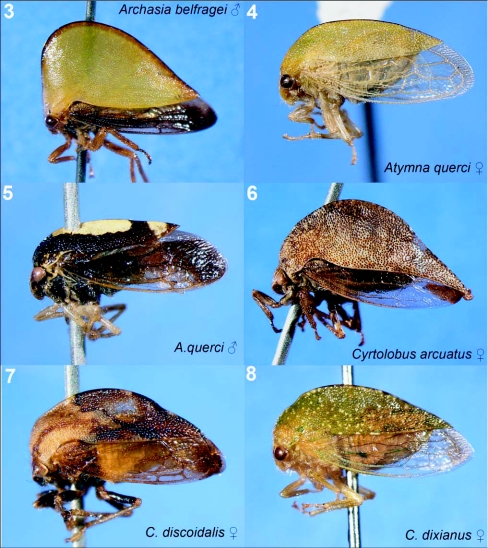
Representative treehoppers of Delaware Water Gap National Recreation Area, I. 3, *Archasia belfragei* (♂). 4, *Atymna querci* (♀). 5, *A. querci* (♂). 6, *Cyrtolobus arcuatus* (♀). 7, *C. discoidalis* (♀). 8, *C. dixianus* (♀). For species with sexual dimorphism, figures of both males and females are shown. Photographs of a single sex of a species indicate little sexual dimorphism except *C. arcuatus* and *C.* *discoidalis,* where only females were collected.

The combined data (Table 2) showing when different treehoppers species were collected on sticky cards over the 3-year period reveals that most were trapped in DWGNRA during mid-June. However, many species of treehoppers were temporally segregated on oak. Further research is needed to help elucidate the reason for this segregation. Detailed field studies may help determine if variation in phenology among species is due to differences in duration of the nymphal stage or due to natural selection to avoid competitive pressure.

**Figures 9–13.**
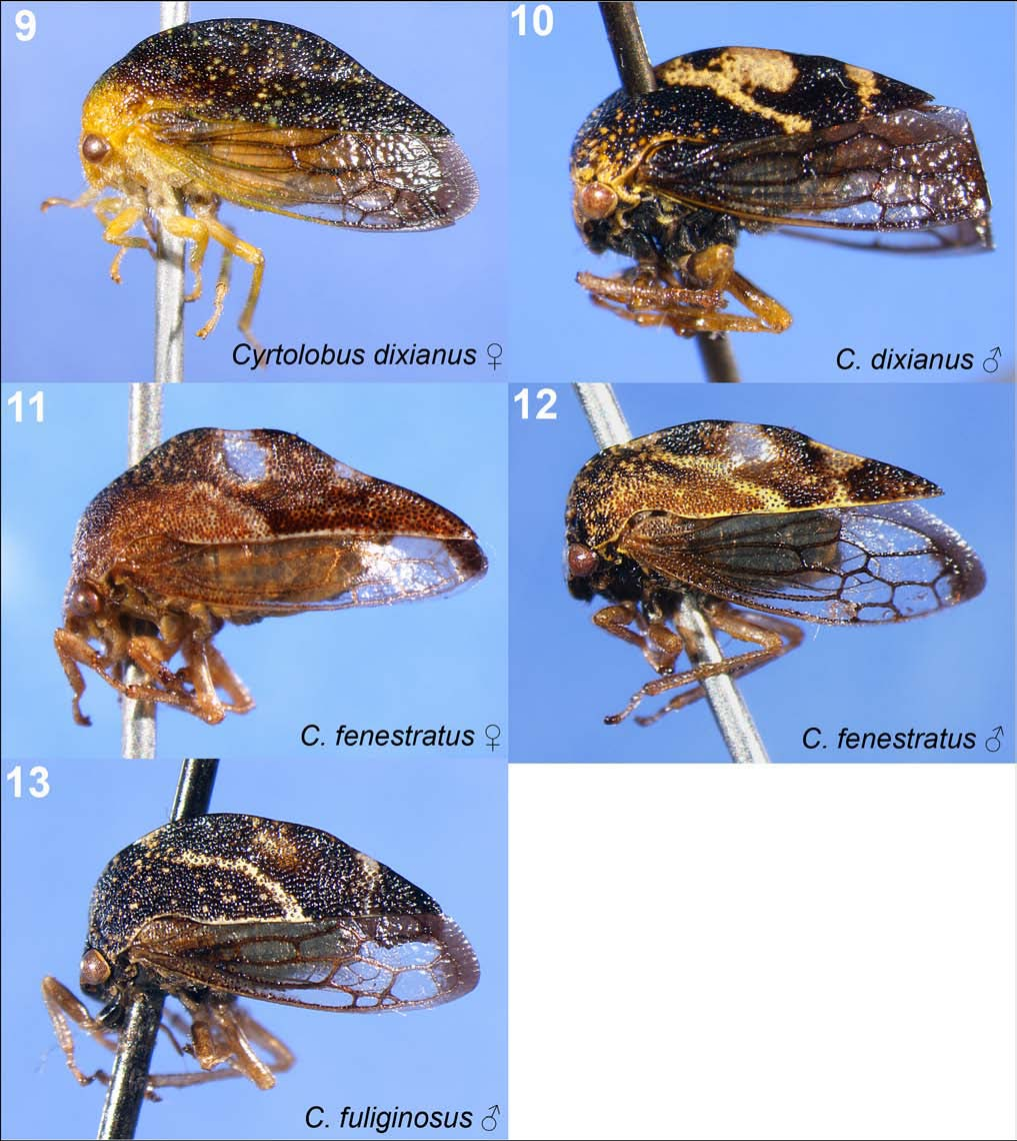
Representative treehoppers, II. 9, *Cyrtolobus dixianus* (♀). 10, *C. dixianus* (♂. 11, *C. fenestratus* (♀). 12, *C. fenestratus* (♂). 13, *C. fuliginosus* (♂). For species with sexual dimorphism, figures of both males and females are shown. No females of *C. fuliginosus* were collected.

A large majority of the specimens collected during all years of the study were male. Frost (1957) also reported larger numbers of males taken at light traps in an oak grove in Centre County, Pennsylvania. Studies using yellow sticky traps to capture leafhoppers (Lessio and Alma 2004) and chrysomelid beetles (Kuhar and Youngman 1995) also showed that males made up a large proportion of the catch. According to Lessio and Alma (2004), the higher capture rate of males on sticky cards was due to the higher dispersal rate of males than females. They also listed other Auchenorrhyncha demonstrating similar behaviors.

**Figures 14–18.**
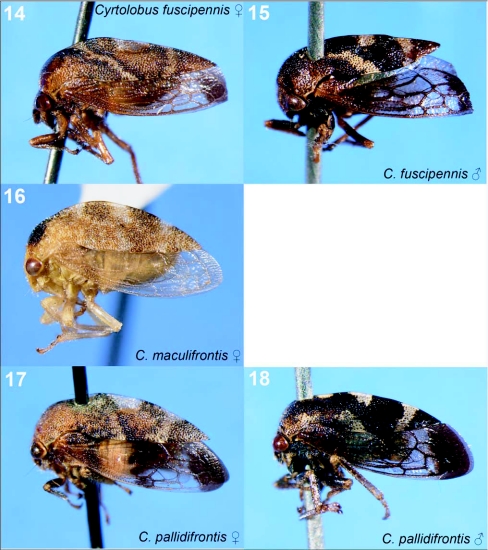
Representative treehoppers, III. 14, *Cyrtolobus fuscipennis* (♀). 15, *C. fuscipennis* (♂). 16, *C. maculifrontis* (♀). 17, *C. pallidifrontis* (♀). 18, *C. pallidifrontis* (♂). For species with sexual dimorphism, figures of both males and females are shown. Only females of *C. maculifrontis* were collected.

Although specimens collected on sticky cards attached to certain oak species do not necessarily indicate a true host, data here may show possible trends. It appears that most treehopper species collected are likely feeding on a variety of oak species. It is likely, however, that certain genera show preferences for either the red (*Ophiderma*) or white oak (*Cyrtolobus*) group. These were the two most common treehopper genera in the collections every year, and they were collected at the same time each year (Figure 2). Their different oak preferences may be a consequence of natural selection to avoid competitive pressure. More specimens were collected from chestnut oak over the 3-year period than any other oak species even though only 13 total trees were used in the study, while the highest species diversity was associated with white oak. Therefore, chestnut oak and white oak may be superb hosts for treehoppers.

**Figures 19–24.**
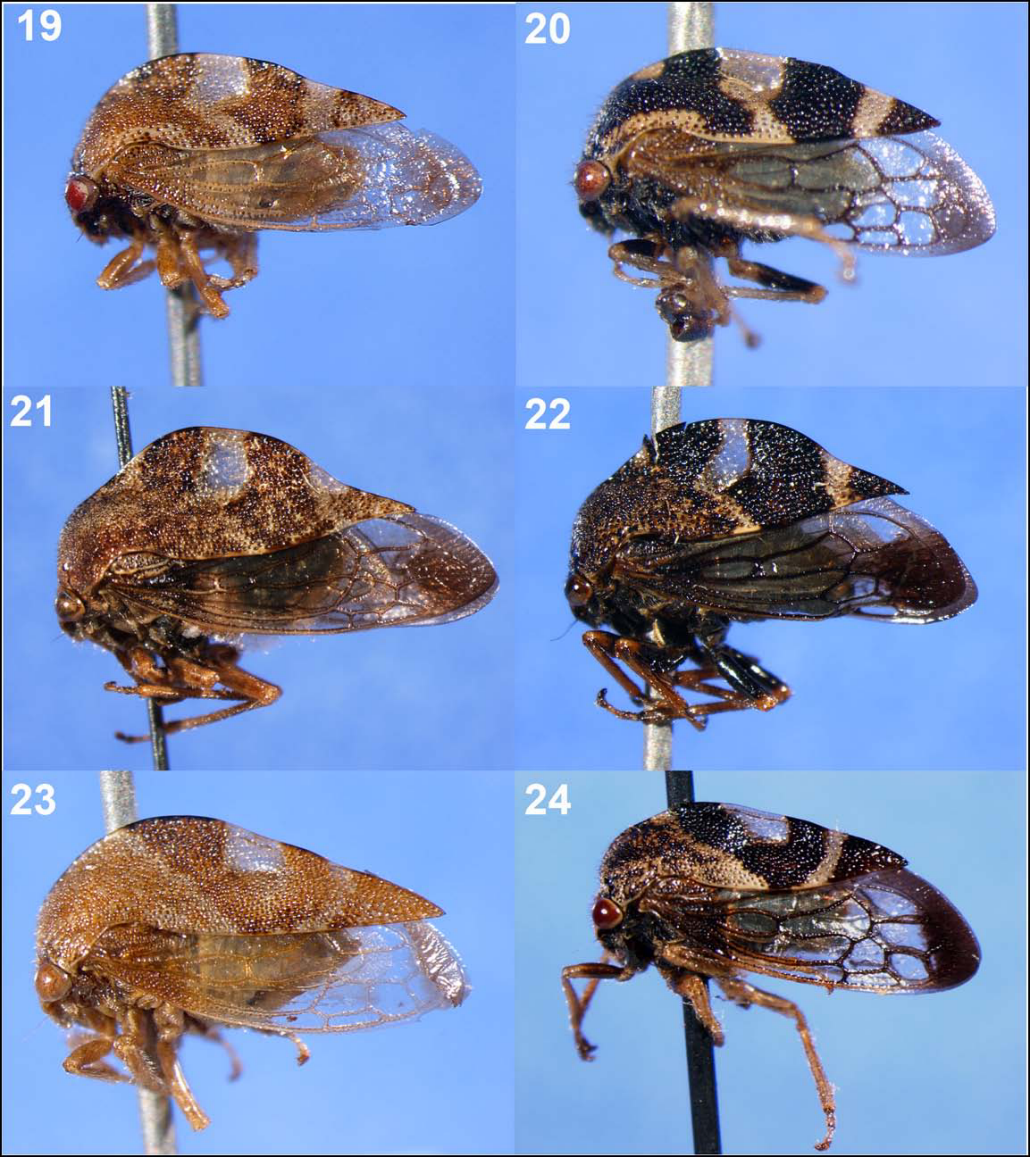
Representative treehoppers, IV. 19, *Cyrtolobus puritanus* (♀). 20, *C. puritanus* (♂). 21, *C. tuberosus* (♀). 22, *C. tuberosus* (♂). 23, *C. vau* (♀). 24, *C. vau* (♂). For species with sexual dimorphism, figures of both males and females are shown.

**Figures 25–30.**
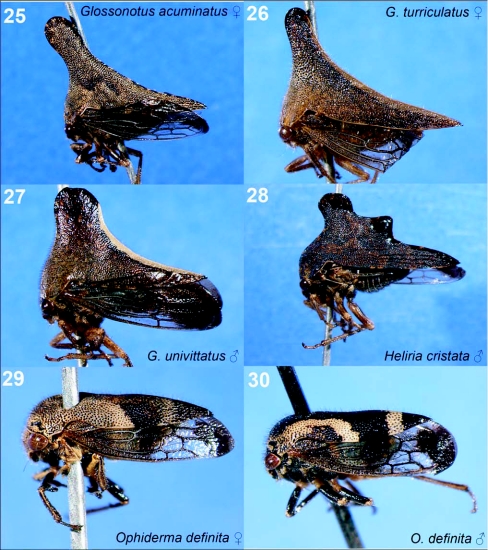
Representative treehoppers, V. 25, *Glossonotus acuminatus* (♀). 26, *G. turriculatus* (♀). 27, *G. univittatus* (♂). 28, *Heliria cristata* (♂). 29, *Ophiderma definite* (♀). 30, *O. definita* (♂). For species with sexual dimorphism, figures of both males and females are shown. Photographs of a single sex of a species indicate little sexual dimorphism.

**Figures 31–36.**
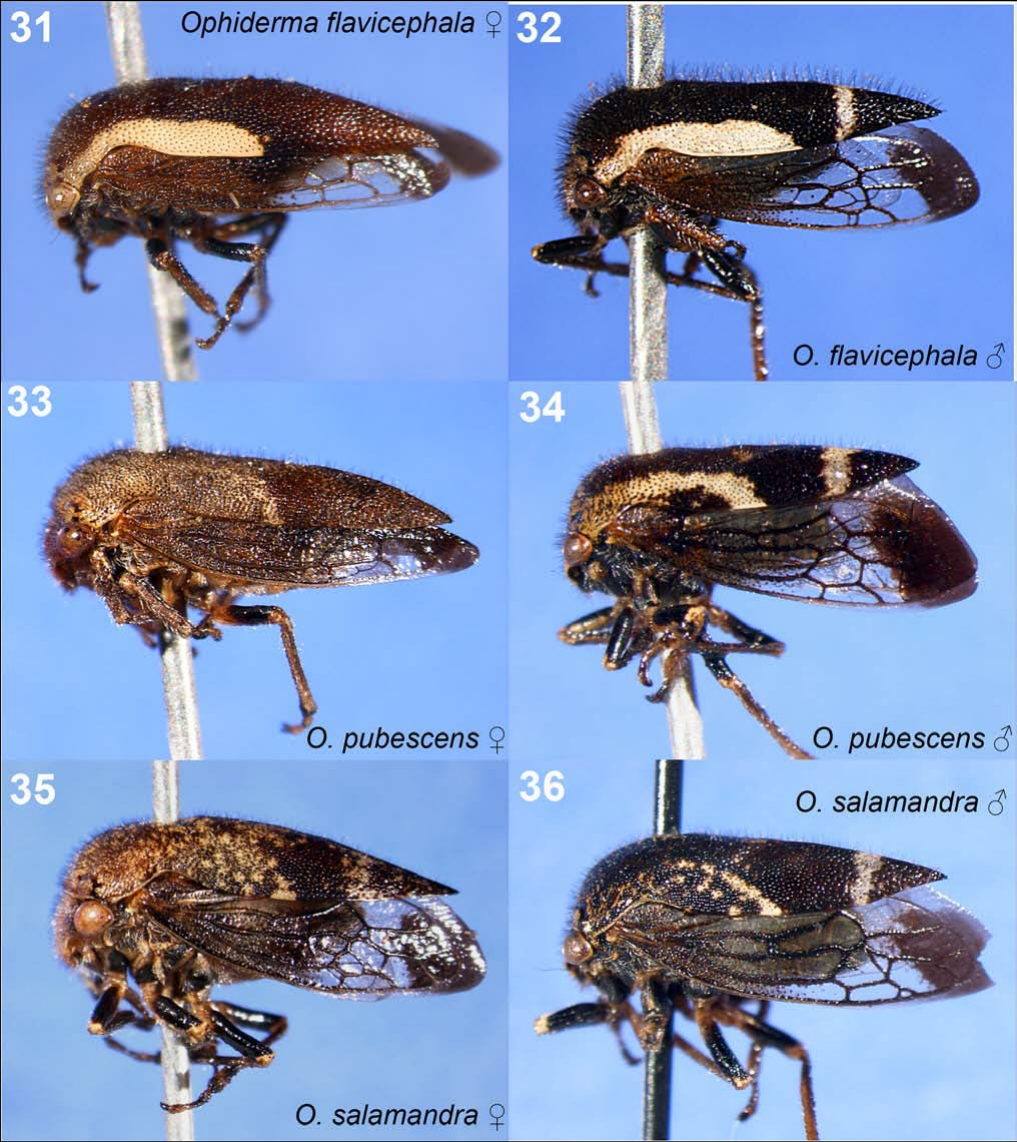
Representative treehoppers, VI. 31, *Ophiderma flavicephala* (♀). 32, *O. flavicephala* (♂). 33, *O. pubescens* (♀). 34, *O. pubescens* (♂). 35, *O. salamandra* (♀). 36, *O. salamandra* (♂). For species with sexual dimorphism, figures of both males and females are shown.

**Figures 37–40.**
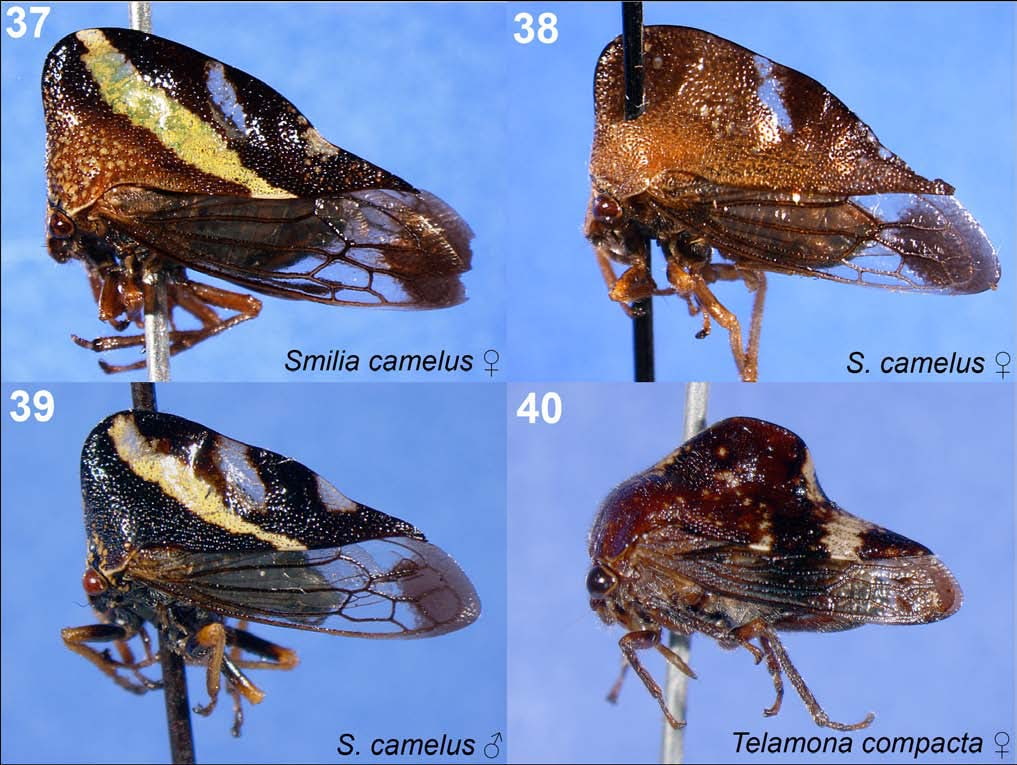
Representative treehoppers, VII. 37, *Smilia camelus* (♀). 38, *S. camelus* (♀). 39, *S. camelus* (♂). 40. *Telamona compacta* (♀). For species with sexual dimorphism, figures of both males and females are shown. Photographs of a single sex of a species indicate little sexual dimorphism.

**Figures 41–44.**
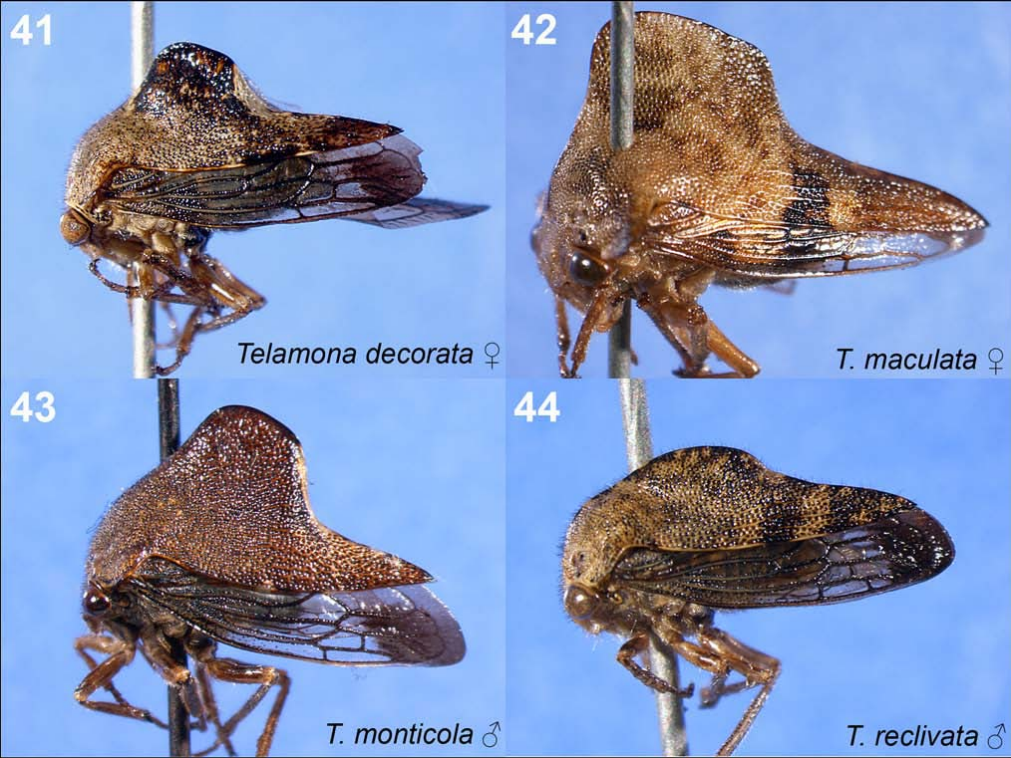
Representative treehoppers, VIII. 41, *Telamona decorata* (♀). 42, *T. maculata* (♀). 43, *T. monticola* (♂). 44, *T. reclivata* (♂). Photographs of a single sex of a species indicate little sexual dimorphism.

**Table 3.**
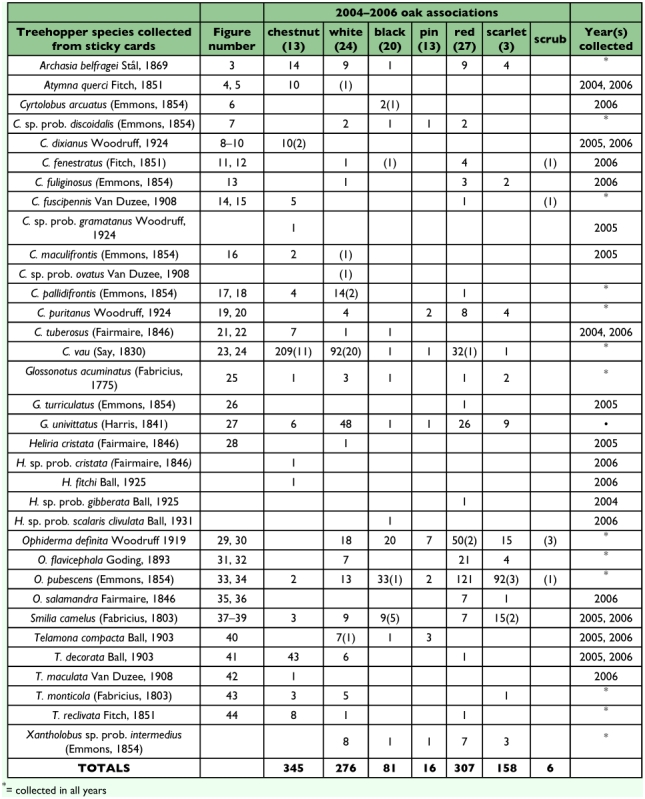
Oak associations and years collected for identified treehopper species from DWGNRA (2004–2006) using yellow sticky cards. The numbers of specimens collected by sweeping/hand-picking are indicated in parentheses. The years collected column only applies to specimens collected on sticky cards. Some of these data were published by Wallace and Troyano (2006) and are included here for comparison.
